# Percutaneous intrabiliary injection of gelatin sponge slurry for the management of acute hemobilia during biliary interventions: an alternative salvage technique

**DOI:** 10.1186/s42155-026-00697-5

**Published:** 2026-07-18

**Authors:** Trieu Hai Pham, Nhat Minh Nguyen, Linh Chi Ho, Trung Quoc Pham, Quyet Ta Nguyen

**Affiliations:** 1 Department of General Surgery, Binh Dan Hospital, Ho Chi Minh City, Vietnam; 2 Department of General Surgery, Pham Ngoc Thach University of Medicine, Ho Chi Minh City, Vietnam; 3 Department of General Surgery, School of Medicine, University of Medicine and Pharmacy, Ho Chi Minh City, Vietnam

**Keywords:** Hemobilia, Gelfoam slurry, Biliary tamponade, Percutaneous biliary intervention, Salvage technique

## Abstract

**Purpose:**

To evaluate percutaneous intrabiliary injection of gelatin sponge (Gelfoam) slurry as a preliminary salvage technique for acute hemobilia during biliary interventions when angiographic embolization is unavailable.

**Material and methods:**

We retrospectively reviewed eight patients with significant hemobilia during percutaneous biliary procedures between September 2024 and December 2025. Notably, seven patients (87.5%) had pre-existing clinical cholangitis that was controlled with therapeutic antibiotics prior to the bleeding event. Gelfoam slurry was injected directly into the biliary tree or tract to achieve hemostasis via internal compression and the promotion of localized thrombosis, while maintaining post-procedural catheter drainage and continued antibiotic coverage.

**Results:**

Immediate technical hemostasis was achieved in all cases (100%). One instance of recurrent bleeding at 10 days was successfully managed with a repeat injection. Post-procedural infectious episodes (fever, leukocytosis) were observed in two patients (25%), requiring an upgrade in targeted antibiotic therapy. The pre-existing infectious baseline in the majority of the cohort complicates the distinction between recurrent cholangitis and de novo foreign-body-related infection.

**Conclusion:**

Preliminary experience suggests that intrabiliary Gelfoam slurry may represent a potential bridging or salvage maneuver for acute hemobilia in resource-constrained scenarios. However, because it relies on internal compression rather than definitive vascular repair, and carries potential risks of infectious exacerbation, it should be applied cautiously with strict follow-up and does not replace definitive transcatheter arterial embolization.

**Graphical Abstract:**

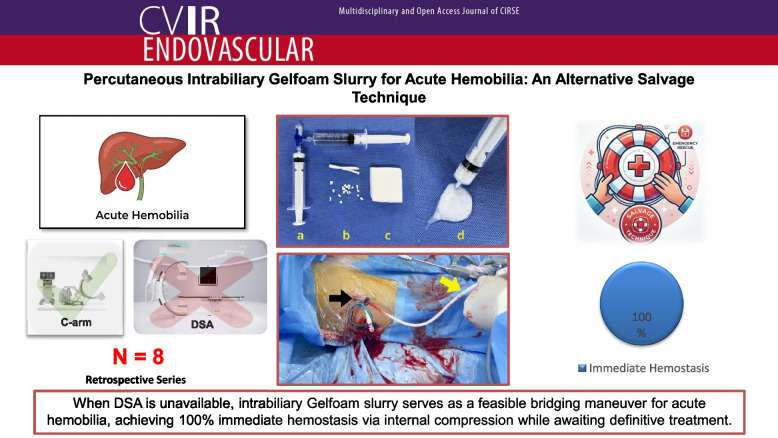

## Introduction

Percutaneous biliary interventions carry a reported 2 to 5% risk of hemobilia [1]. While minor bleeding is often self-limiting, severe hemorrhage demands urgent management to prevent hemodynamic instability [1, 2]. Although transcatheter arterial embolization (TAE) is the gold standard, immediate access to Digital Subtraction Angiography (DSA) is not always feasible in C-arm operating theaters. Additionally, identifying the bleeding source is challenging when hemorrhage originates from angiographically occult portal or hepatic veins [3].

Gelatin sponge (Gelfoam) is widely used for tract plugging for hemostasis [4, 5], yet its direct intrabiliary application for hemostatic tamponade remains rare, with limited data mostly restricted to endoscopic approaches [6]. We report an alternative salvage technique using percutaneous intrabiliary injection of Gelfoam slurry to control acute hemobilia in eight patients where immediate angiography was unavailable, or the bleeding source was indeterminate. We emphasize that this technique is not intended to replace definitive TAE, but rather serves as a temporary bridging maneuver in selected, resource-constrained scenarios.

## Materials and methods

### Study design and patient selection

This retrospective series analyzed eight patients experiencing acute hemobilia during ultrasound and C-arm-guided biliary interventions between September 2024 and December 2025, including percutaneous transhepatic biliary drainage (PTBD, *n *= 2), percutaneous transhepatic cholangioscopic lithotripsy (PTCSL, *n *= 4), and stenting (*n* = 2). Prior to the index procedures, baseline coagulation profiles (INR and platelet counts) were routinely assessed. Patients included in this series had INR ranging from 1.00 to 1.26 and platelet counts between 92 and 460 K/uL, ensuring no severe uncorrected coagulopathy influenced the bleeding events.

Inclusion criteria were defined as follows:

- Immediate onset of hemobilia during the procedure, manifested by hemodynamic instability or fresh blood output via the sheath/catheter.
- Unavailability of immediate DSA or an indeterminate bleeding source under fluoroscopy.

### Rationale for the salvage technique

**Fig. 1:**
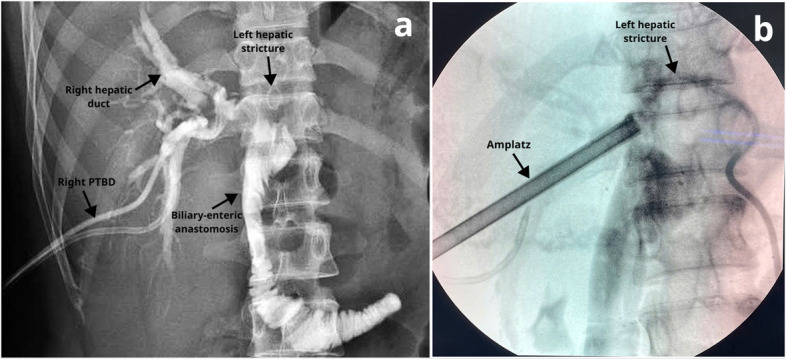
C-arm fluoroscopy (Case 6). **a** Right-sided cholangiogram demonstrating a left hepatic stricture. **b** Acute hemobilia triggered during attempted stricture navigation via a right-approach Amplatz sheath

Hemobilia may originate from various vascular sources, including the hepatic artery, portal vein, or hepatic veins. In the operating room setting, utilizing only C-arm fluoroscopy, identifying the exact vessel—particularly arterial sources—is technically challenging compared to DSA (Fig. 1).

We adapted the Gelfoam slurry technique for intrabiliary use based on three key factors [4]:

- Dual-action hemostatic mechanism: instead of acting merely as a fluid tamponade, Gelfoam achieves hemostasis through two synergistic effects. First, by absorbing up to 45 times its weight in blood, the slurry expands within the confined space of the biliary tract, exerting direct internal mechanical compression against the bleeding vessel [6, 7]. This mechanical stasis abolishes the active high-velocity flow of the hemorrhage. Second, the gelatin matrix acts as a robust scaffold for platelet aggregation, promoting rapid and durable localized thrombosis at the site of vascular injury [7, 8].
- Safety profile: the material is biodegradable, resorbing within 3 to 5 weeks. The risk of transient biliary obstruction is acceptable given the life-threatening nature of the hemorrhage [3].
- Literature precedent: this approach adapts principles established in percutaneous tract embolization and rare reports of endoscopic (endoscopic retrograde cholangiopancreatography—ERCP) injection [6].

### Slurry volume, injection technique, and post-operative management

#### Slurry preparation (Fig. 2)

**Fig. 2:**
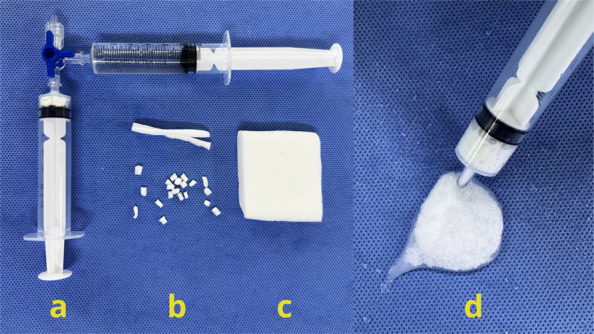
Gelfoam setup showing syringes connected by **a** 3-way valve, **b** prepared Gelfoam cut, **c** intact Gelfoam, and **d** the resulting Gelfoam slurry ready for use

Sterile gelatin sponge sheets were cut into 1–2 mm particles and mixed with saline and iodinated contrast in a 10-mL syringe. The mixture was agitated vigorously (20–30 times) between two syringes via a three-way stopcock to create a homogenous flowable slurry [3].

#### Injection procedure

**Fig. 3:**
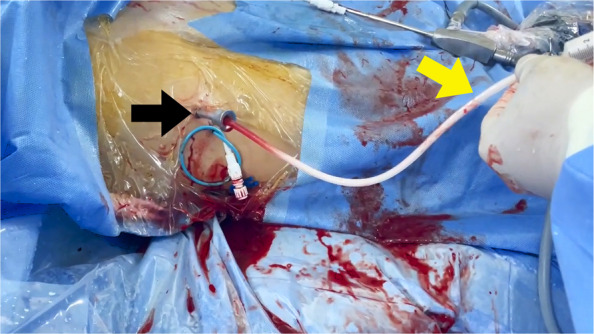
The injection of Gelfoam slurry (yellow arrow) into the bleeding percutaneous intrabiliary via the existing Amplatz sheath (black arrow)—case 6

Upon recognition of refractory hemobilia, the existing biliary catheter or sheath was used as the conduit. The Gelfoam slurry was injected at a standard volume of 10 mL per attempt, administered at a controlled, steady rate of 0.5 to 1 mL per second to gradually build intrabiliary pressure without causing abrupt ductal injury (Fig. 3). If active bleeding persisted fluoroscopically or clinically after the initial administration, the injection was repeated up to a maximum of three attempts.

#### Catheter clamping and drainage

Following the injection, the catheter was temporarily clamped (capped) for exactly 30 min to maximize the internal compression effect and allow sufficient time for stable clot formation. We recognize that temporarily clamping the biliary tree theoretically elevates intrabiliary pressure and increases the risk of cholangitis or bacteremia. To mitigate this infectious complication, optimal safety is achieved in patients who have multiple (2 to 3) biliary drainage catheters in place. This multi-drain setup ensures that while the index bleeding tract is temporarily clamped for hemostasis, the overall biliary tree remains adequately decompressed.

#### Antibiotic therapy

Regarding the antibiotic protocol, only one patient (Case 3) received standard prophylactic broad-spectrum antibiotics strictly for the index procedure. The remaining seven patients presented with clinical evidence of cholangitis 4 to 5 days prior to the intervention. These seven patients were already receiving targeted therapeutic intravenous antibiotic regimens (e.g., Piperacillin/Tazobactam, Ertapenem, or Cefoperazone/Sulbactam), which successfully controlled the clinical signs of infection and normalized inflammatory markers prior to the biliary procedure.

Following the intrabiliary Gelfoam injection, all patients continued their respective antibiotic therapies (either prophylactic converted to therapeutic, or continuing the existing therapeutic regimen) to strictly mitigate the risk of cholangitis associated with intraluminal foreign material. Additionally, to prevent catheter occlusion by Gelfoam particles or blood clots while preserving the newly formed thrombus, aggressive catheter flushing was strictly avoided in the initial 24–48 h. After 48 h, if there was an absence of spontaneous bile drainage, the catheter was gently flushed with 5–10 mL of normal saline (0.9% NaCl) once daily to maintain patency. Patients were closely monitored during this process.

## Results

### Patient characteristics and clinical presentation (Table 1)

**Table 1:** Characteristics, procedural details, and outcomes of the eight patients

Case	Age/ sex	Diagnosis	Index procedure	Coagulation (INR/PLT*)	Slurry volume (mL)	Antibiotic setting	Follow-up durations	Post-procedural outcome and follow-up
1	35/male	Hepatolithiasis	PTBD dilation (Left)	1.17/460	10	Therapeutic	6 months	Immediate hemostasis. Cholangioscopy at 3 weeks confirmed complete Gelfoam dissolution
2	63/male	Klatskin tumor	PTBD exchange	1.25/123	30	Therapeutic	3 months	Immediate hemostasis. Subsequently underwent successful metallic stenting
3	66/male	Bilateral hepatolithiasis	PTCSL (Lithotripsy and stricture dilation)	1.05/140	30	Prophylactic	1 month	Immediate hemostasis. Developed fever/leukocytosis at 7 days; resolved with antibiotics. Cholangioscopy at 3 weeks showed no residual Gelfoam
4	50/female	Right posterior intrahepatic stones (Post-robotic Whipple)	PTCSL	1.02/92	20	Therapeutic	14 months	Immediate hemostasis; hemodynamics stabilized. Clinical stability maintained during follow-up
5	62/male	Biliary Stones	PTCSL	1.12/290	10	Therapeutic	2 months	Immediate hemostasis. Clinical stability maintained
6	29/female	Left hepatolithiasis and stricture	PTCSL (Right approach); Stricture dilation via Amplatz	1.00/373	20	Therapeutic	1 month	Immediate hemostasis. Follow-up endoscopy confirmed complete Gelfoam dissolution
7	55/male	Mucinous cholangiocarcinoma	Stent placement	1.18/277	30	Therapeutic	1 month	Immediate hemostasis. Died at 1 month due to advanced cancer progression (no recurrent bleed)
8	67/male	Malignant obstruction	Stent placement (left)	1.26/298	30	Therapeutic	1.5 months	Initial hemostasis. Concurrent infection and recurrent bleeding at 10 days; managed with antibiotic upgrade and a repeat injection (30 mL). Resolved

^*^PLT (platelets) measured in 10^3^/μL (K/μL)

The study included eight patients (6 males, 2 females; mean age 53.4 years) experiencing hemobilia during PTBD (*n *= 2), PTCSL (*n *= 4), or stenting (*n *= 2). Clinical severity varied: one patient developed hemorrhagic shock requiring resuscitation, another required transfusion for hemoglobin drop, and one presented with a pre-existing portal-biliary fistula.

### Outcomes

Technical success, defined as the immediate angiographic or clinical cessation of bleeding, was achieved in all 8 patients (100%). Following the intrabiliary injection of the Gelfoam slurry, hemostasis was confirmed by the absence of fresh blood output via the sheath or drain.

### Follow-up and safety profile

The median follow-up time for the entire cohort was 2 months (range 1–14 months). Technical success, defined as immediate hemostasis, was achieved in 100% of cases. However, regarding the safety profile and delayed complications, infectious episodes were recorded post-procedurally. Case 3 developed a fever (39 °C) and leukocytosis (WBC 11.68 K/μL) at 7 days post-procedure, which resolved with targeted antibiotic therapy. Case 8 experienced a concurrent infection (WBC 41 K/μL) and recurrent bleeding 10 days post-procedure, requiring an upgrade in antibiotic therapy (Ertapenem) and a repeat Gelfoam injection, which ultimately resolved the hemorrhage.

## Discussion

While TAE is the gold standard for severe hemobilia, its utility is limited in resource-restricted settings lacking immediate DSA access [3]. Our preliminary experience indicates that percutaneous Gelfoam slurry may represent a potential salvage option, achieving immediate hemostasis in all cases in our series. However, these findings must be interpreted cautiously given the limitations of a small retrospective cohort and short follow-up period.

We hypothesise that the mechanism relies on internal mechanical compression and platelet aggregation to facilitate localized thrombosis, rather than simple fluid tamponade, although this behavior has not been specifically demonstrated in the biliary system [4, 7, 8]. This is optimized by injecting 10 mL of slurry (0.5–1 mL/s) and clamping the drainage catheter for 30 min to maximize stable clot formation. This technique is highly advantageous under C-arm fluoroscopy when identifying the precise bleeding source is infeasible.

Although Gelfoam is established for tract plugging, its intrabiliary application is novel, extending the endoscopic experience described by Wang et al. to percutaneous interventions [6].

Regarding the safety profile, deliberately inducing biliary stasis via clamping and intrabiliary injection carries theoretical risks of exacerbating infectious complications (such as cholangitis) and temporarily masking an ongoing vascular injury. We mitigated these risks through post-procedural catheter drainage and continuous therapeutic antibiotic coverage. The risk of masking an underlying injury was exemplified in Case 8, where secondary hemorrhage occurred at 10 days, likely due to Gelfoam biodegradation combined with a concurrent local infection exposing the unhealed defect [4].

While Gelfoam typically biodegrades in 3–5 weeks via phagocytosis, its exact breakdown profile in the biliary tract remains undefined. The lack of ductal macrophages could delay clearance; however, detergent bile salts and proteolytic enzymes from concurrent cholangitis may accelerate gelatin matrix degradation. Clinically, our cholangioscopic follow-up confirmed complete macroscopic dissolution at 3 weeks, suggesting that the physiochemical properties of bile and restored antegrade flow are sufficient for material clearance.

Limitations include the small retrospective design, lack of a control group, and absence of systematic endoscopic surveillance to confirm long-term material resorption. Therefore, this technique should not replace selective angiographic embolization. Rather, it serves as a temporary bridging maneuver reserved for crisis management when definitive treatment is inaccessible.

## Conclusion

Preliminary experience indicates that percutaneous intrabiliary Gelfoam slurry may represent a potential salvage option for acute hemobilia when immediate angiography is unavailable. While effective for initial hemostasis, it does not replace definitive embolization due to the risks of delayed recurrence and cholangitis. Its judicious application mandates strict biliary decompression and continuous therapeutic antibiotic coverage. Further prospective validation is required to establish its long-term safety profile.

## Data Availability

The datasets generated and/or analyzed during the current study are available from the corresponding author on reasonable request.

## Abbreviations

TAE: Transcatheter arterial embolization
DSA: Digital subtraction angiography
PTBD: Percutaneous transhepatic biliary drainage
PTCSL: Percutaneous transhepatic cholangioscopic lithotripsy
ERCP: Endoscopic retrograde cholangiopancreatography
PLT: Platelets
